# P02.24. Reducing stress and cultivating well being in educators and parents with special needs children: effects of a mindfulness training program

**DOI:** 10.1186/1472-6882-12-S1-P80

**Published:** 2012-06-12

**Authors:** R Benn, T Akiva, S Arel, R Roeser, J Eccles

**Affiliations:** 1 University of Michigan, Ann Arbor, USA; 2 Portland State University, Portland, USA

## Purpose

Parents and teachers of children with developmental challenges and special learning needs face unique social-emotional challenges in caregiving. Stress associated with their roles has been shown to impact parents’ and special educators’ health and well-being, as well as the quality of their parenting and teaching. In this paper, we report results of a pilot study conducted to investigate the efficacy of mindfulness based training program (MT) in facilitating improvement in caregiver mental health. Using a RCT waitlist design, 70 parents and educators of children with special needs were randomized to participate in a 9 session, 36 hour group-based MT program called SMART (Stress Management and Relaxation Techniques). This program, based on MBSR, included content on emotion theory and regulation, forgiveness and compassion, and application practices specific to caregiving/teaching.

## Methods

Participants completed a battery of standardized measures at three time points: baseline, program completion and two months follow-up. The measures included several indicators of mindfulness, distress, positive affect, compassion, well being, and caregiving competence. In addition, we explored the impact of participant demographic and lifestyle and characteristics (e.g., marital status, previous history of meditation experience, engagement in self-care practices and religious observance), health status, and role (teacher vs. parent). The influence of several intervention parameters on outcomes were also examined within the MT group (e.g. number of sessions attended, number of minutes of average weekly mindfulness practice and type of format program participation( twice a week vs weekly format).

## Results

Study findings provide strong preliminary evidence for the benefits of MT in reducing stress and anxiety, improving mindfulness and psychological well-being, and facilitating hope and gratitude in both parents and teachers. Medium to large effect sizes were shown on several measures upon program completion and at follow-up.

## Conclusion

MT is an effective intervention for facilitating well-being in teachers and parents of children with special needs whether delivered in weekly or biweekly program format.

